# A short review of graphene in the microbial electrosynthesis of biochemicals from carbon dioxide

**DOI:** 10.1039/d2ra02038f

**Published:** 2022-08-15

**Authors:** L. F. Chen, H. Yu, J. Zhang, H. Y. Qin

**Affiliations:** New Energy Materials Research Center, College of Materials and Environmental Engineering, Hangzhou Dianzi University Hangzhou 310018 China chlf@hdu.edu.cn +86 571 86878609

## Abstract

Microbial electrosynthesis (MES) is a potential energy transformation technology for the reduction of the greenhouse gas carbon oxide (CO_2_) into commercial chemicals. The major bottlenecks in the development of highly productive MES systems are the low bacterial loading, low electron transfer rate and low production of relevant chemicals, which limit the future potential for scaling up this process. Graphene has excellent electrical conductivity, remarkably high carrier mobility, special intrinsic mechanical strength, chemical stability, outstanding specific surface area, and biocompatibility. Therefore, in this regard, graphene can overcome these challenges and provide new opportunities. Graphene is suited for use as a cathode for increasing the bacterial loading and boosting the performance of MES. Over the last decade, graphene has been extensively developed and explored in MES. Graphene incorporation in cathodes can augment the surface area, reduce the resistance, and increase the electron transfer rate; thus, high current density, high coulombic efficiency, and high chemical production can be achieved. To better understand and further explore the modification of graphene-based materials as cathodes in MES systems, it is quite necessary to review and summarize recent developments in this field. Therefore, in this report, we briefly survey and discuss the up-to-date research activities regarding graphene in cathode modification and fabrication, with particular emphasis on their fabrication strategies and characterization, highlighting their key roles in MES systems, as well as presenting the challenges and the future prospects.

## Abstract 2

Microbial electrosynthesis (MES) is a potential energy transformation technology for the reduction of the greenhouse gas carbon oxide (CO_2_) into commercial chemicals.

## Graphical abstract

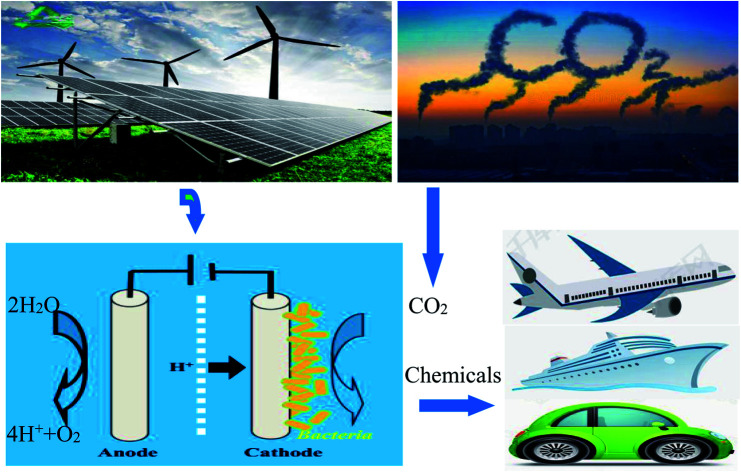

## 1. Background

**Fig. 1:**
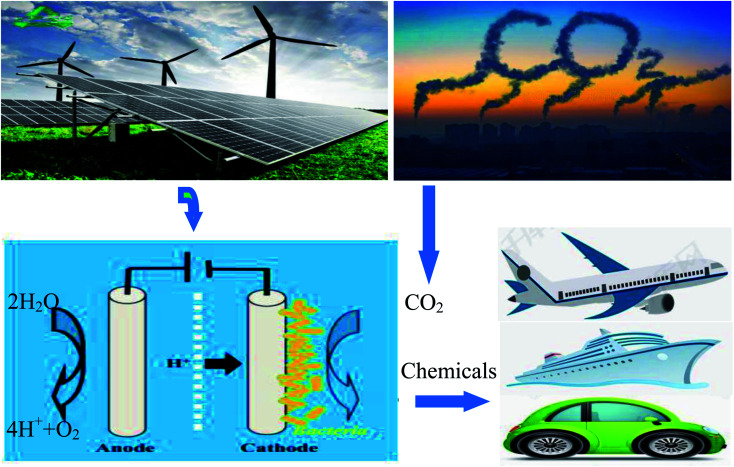
A diagram of CO_2_ capture by the microbial electrosynthesis of biochemicals from renewable energy and chemical energy utilization.

Due to the rapid development of human civilization and the economy, the increasing demand for energy has mankind facing huge challenges in the 21st century. The dependence on fossil fuels and continuous growth in annual carbon oxide (CO_2_) emission have resulted in global warming and climate change issues.^1^ Therefore, the reduction of the CO_2_ concentration in the atmosphere has become a big problem and priority. CO_2_-capturing technologies and a replacement for fossil energy sources with sustainable energy systems are necessary.^2,3^ The rapid development of green energy technologies such as renewable energy has become a research focus in recent years.^4,5^ Microbial electrochemical synthesis is a novel strategy in which electroactive microorganisms use electrons obtained from solid electrodes to convert waste organic matter or CO_2_ into organic compounds and store them in the form of chemical energy.^6,7^ Microbial electrochemical synthesis is an attractive method to generate renewable fuels, and is perhaps the most promising way to meet future human energy demand.^8–10^ The electrochemical synthesis of microorganisms can reduce the greenhouse effect and eliminate CO_2_ emissions, which can minimize the CO_2_ content in the atmosphere.^11–13^ By converting abundant and inexpensive CO_2_ into valuable chemicals, we can create a new sustainable energy transformation system. Microbial products synthesized with CO_2_ have the following advantages: they do not rely on limited arable land and precious water resources, and they are virtually non-toxic and harmless to living organisms. If the electrical energy required for microbial electrochemical synthesis comes from photovoltaic power generation systems, MES may represent an artificial form of photosynthesis with high efficiency.^14^ Microorganisms can use solar energy to convert CO_2_ and water into organic compounds with oxygen as a byproduct, and solar energy can thus be stored in the form of chemical energy. Organic matter can provide fuel for transportation or it can be extracted and used at any time when urgently needed within existing infrastructures. A diagram of CO_2_ capture by the microbial electrosynthesis of biochemicals from renewable energy and chemical energy utilization is shown in Fig. 1.^15^

MES is a prospective energy transformation technology in which the greenhouse gas CO_2_ can be reduced into commercial chemicals by electroactive microorganisms with electrons obtained from the cathode of a bioelectrochemical reactor.^16–18^ The conversion of CO_2_ into valuable organic compounds such as acetate and methane was first reported in 1995.^19^ Subsequently, the synthesis of acetate from CO_2_ by acetogenic microorganisms such as *Sporomusa* and *Clostridium spp.* was presented.^29,30^ Besides acetate, other chemicals including formate, butyrate, ethanol, isopropanol, and butanol can be obtained in CO_2_-fed bioelectrochemical reactors.^20–23^ Since then, considerable research efforts have been made to increase the production rates for greater potential scaling up of such systems.^24^ For MES reactor optimization, all the reactor components, including activation of the microbial catalyst, augmentation of the cultivation medium composition, improving the reactor design, and spatial arrangement of the cathode to enhance its interaction with the microorganism cathodes, have been studied in recent years.^25,26^ The core role of MES is electron transfer from the cathode to microbes. Thus, the cathode plays a key role by donating electrons to the microbes in the process of MES CO_2_ reduction.^27^ Therefore, many strategies have been employed to develop novel electrode materials and spatial arrangements.^28^ For the construction of efficient cathodes, the ideal materials should possess good biocompatibility, high catalytic activity, low charge transfer resistance, high surface area, high durability, and low production cost.

**Fig. 2:**
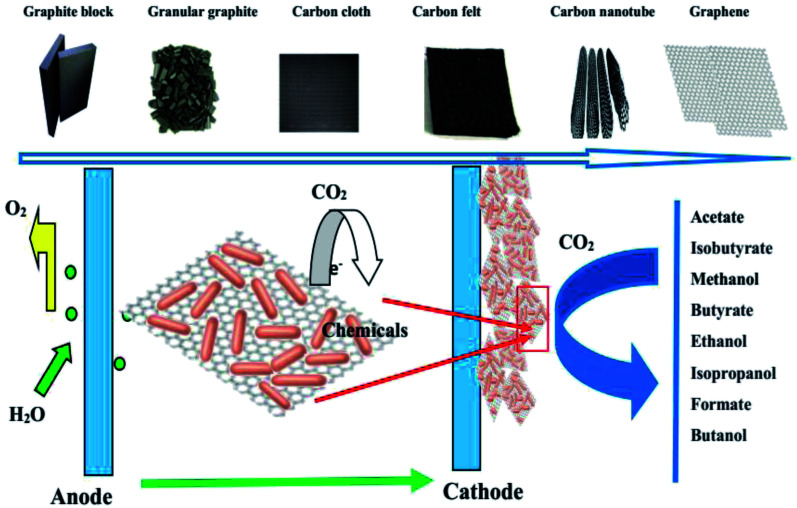
A schematic of the MES cathode development history and the various related biochemicals.

In early cathode research on MES systems, a negatively charged solid-state graphite block was used as the electron donor source.^29,30^ Due to its low porosity and low accessible surface area resulting in limited microorganism adherence, it was difficult to achieve higher productivity. Granular graphite as a cathode was then proposed, and a high volumetric acetate production rate from CO_2_ was obtained due to its high volumetric surface area and porosity.^31,32^ In the past several years, carbon cloth and carbon felt have been widely used because of their high reactive surface area and high porosity, chemical stability, and better electrical conductivity, biocompatibility, and flexibility compared to previous electrodes.^33,34^ Recently, combinations of carbon felt, carbon cloth, and carbon nanomaterials such as carbon nanotubes (CNTs) and graphene have been explored in MES systems.^35–38^ A schematic of the MES cathode material development history and the various related biochemicals produced is shown in Fig. 2.

Graphene is a two-dimensional atomic lattice structure in which carbon atoms are arranged in an sp^2^-bonded hexagonal pattern.^39^ It is currently known as the thinnest material in the world. Ever since it was discovered in 2004,^40^ studies on graphene quickly became an international hot topic.^41,42^ Particularly in the last decade, it has been extensively studied in the scientific and engineering fields.^43,44^ Owing to its distinctive properties, graphene has excellent electrical conductivity, remarkably high carrier mobility, special intrinsic mechanical strength, chemical stability, outstanding specific surface area, and biocompatibility. Due to these special characteristics, in recent years, graphene has also been extensively studied and explored in the environmental, medical and electrochemical fields, especially in bioelectrochemical systems.^45^ In order to better understand and outline future prospects for the modification of graphene and graphene-based composite/hybrid material cathodes in MES systems, it is quite necessary to review and summarize recent developments in this field. Therefore, in the present report, we briefly discuss and outline the up-to-date research activities on graphene in the modification and fabrication of microbial electrosynthesis cathodes, with particular emphasis on their fabrication strategies and characterization, highlighting the key roles of graphene in MES systems, and finally the challenges faced and their future prospects.

## 2. Strategies

**Fig. 3:**
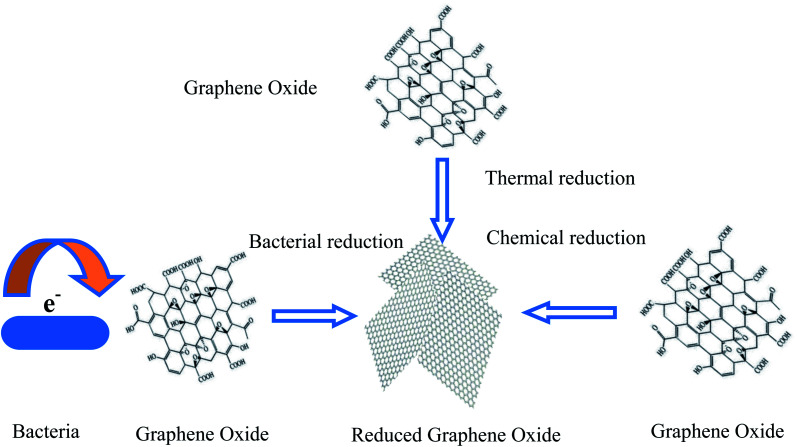
Schematic methods for constructing reduced graphene oxide.

Due to its high surface-to-volume ratio, high electrical conductivity, chemical stability, and biocompatibility, graphene is suited to modifying cathodes for the promotion of MES productivity and scalability. Recently, graphene and graphene-based composite/hybrid materials have been used to modify the cathodes of MES, which has resulted in a significant improvement in chemical production. Using graphene for the modification of cathodes, the performance of bacterial attachments, biofilm development and the interaction between microorganisms and the electrode can be improved. The construction of partial graphene-based cathodes uses the starting material of graphene oxide (GO).^46^ Through GO reduction to reduced graphene oxide (RGO), RGO can be decorated on the cathode. GO reduction methods can be used, such as thermal annealing, chemical reducing agents, and bacterial reduction such as self-assembly (Fig. 3). Furthermore, for the MES cathodes modified by graphene, chemical synthesis strategies are also usually adopted to construct graphene or graphene-based composite/hybrid cathodes. Thus, the cathode building strategies including direct GO reduction, self-assembly, and the synthesis of graphene-based composites or hybrids *via* a two-step chemical method, and the typical experiments are illustrated herein.

### 2.1. Direct GO reduction

**Fig. 4:**
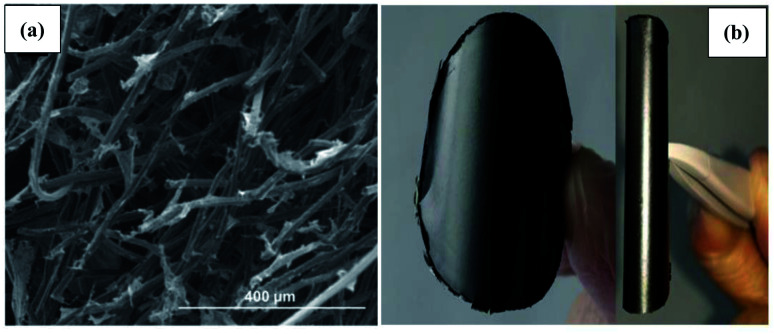
(a) High-magnification SEM image of the 3D-G-CF cathode and (b) bent RGO paper to demonstrate its mechanical flexibility. (a) Adapted/reproduced from ref. 47 with permission from Elsevier Ltd, copyright 2016. (b) Adapted/reproduced from ref. 48 with permission from Springer Nature, copyright 2017.

Graphene easily restacks and effortlessly forms irreversible agglomerates due to the van der Waals forces between its sheets. Meanwhile, the hydrophobicity of graphene can restrict its dissolution in water. Therefore, it is not easy to directly modify graphene sheets on cathodes. Hence, the modification strategies are often based on the water solubility of GO. GO has hydrophilic properties due to its multiple carboxyl, epoxy, carbonyl, and hydroxyl functional groups. Owing to its water solubility, GO can exist as a single layer in water. Thus, GO layers can be impregnated on cathode materials such as carbon cloth or carbon fiber. After the reduction process, RGO can be *in situ* fixed on the cathode materials, and the surface of the cathode can be enhanced. The chemical reducing agents used include sodium borohydride, ascorbic acid, and hydrazine. For example, a very simple method involves dripping GO on the carbon felt surface and then directly reducing it to RGO using the reducing agent of sodium borohydride.^80^ The larger specific surface area of RGO infiltrated into the carbon cloth lattice can enrich microorganism attachment and result in the high production of acetate and butyrate. Using ascorbic acid as a chemical reducing agent, a carbon felt cathode soaked with GO was directly reduced to RGO *via* a solvothermal reduction process.^47^ Using scanning electron microscopy (SEM) (Fig. 4a), it can be seen that RGO nanosheets were *in situ* grafted on the carbon fiber. The high specific surface area and the high conductivity of RGO are beneficial to the attachment of bacteria and the electron extracellular transfer rate. The reduction degree of RGO is related to the chemical reducing agent. For the same reducing agent, a low reduction degree can result in high residual oxygen functional groups. Therefore, the as-obtained RGO has high resistance, which brings about a low electron transfer rate and low chemical production. A high degree of reduction can result in high electron mobility and high mechanical strength; therefore, the RGO-based cathode has high performance for chemical production. The amount of RGO sheets on CF can also influence the morphology of the 3D-graphene CF cathode. By increasing the amount of RGO, the hierarchical porous structure of 3D-graphene CF can be formed. The porous structure can increase the total cathode surface area and the specific surface area can be increased greatly. The porosity of 3D-graphene CF can enhance the colonization of microbes as well as increase the diffusion of CO_2_ and promote the transfer of the substrate and nutrients.

In addition to its infiltration into and immobilization on the cathode of MES *via* the reduction of GO sheets to RGO, GO can be prepared as a paper and then directly reduced and fabricated as freestanding and flexible RGO paper, which can then be exploited as a novel bioelectrochemical cathode to enhance electron transfer between the cathode and microbe.^48^ As shown in Fig. 4b, RGO paper exhibited high flexibility, and can be bent or rolled. Due to these excellent properties, RGO paper can be used to substantially increase the electrode surface area available for electronic interactions with microbes. The surface morphology of graphene on RGO paper effectively did not change with increasing thickness. The surface area availability of graphene only comes from the outer layer of RGO paper and only graphene on the outer layer of RGO paper can have direct electronic interactions with the microbes in the MES reactor. The internal layer of graphene cannot be used well and only acts as a conductive layer. Thus, the performances of MES reactors equipped with difference thicknesses of RGO paper can be almost unchanged.

### 2.2. Self-assembly

**Fig. 5:**
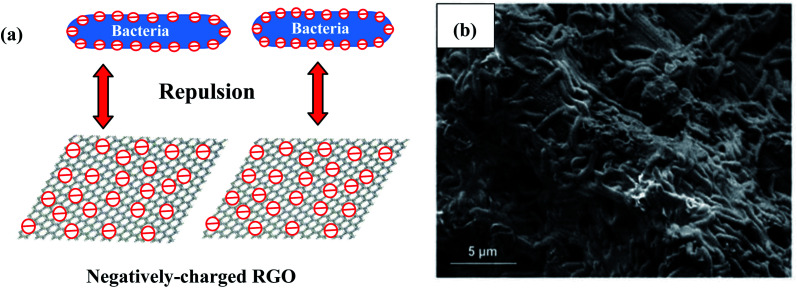
(a) A model of electrostatic repulsion between negatively charged bacteria and RGO. (b) SEM image of the RGO-TEPA-CC cathode in the MES reactor driven by the *S. ovata* wild type. (b) Adapted/reproduced from ref. 54 with permission from The Royal Society of Chemistry, copyright 2016.

Strong bacterial affinity with the electrode is favors good adhesion, increases the electron transfer rate, and improves the overall system properties. It has also been reported that it is a disadvantage that graphene is negatively charged, since this can limit its electrostatic interaction with the negatively charged surface of bacterial cells in BESs (Fig. 5a).^49,50^ In microbial electrosynthesis, electrostatic repulsion can inhibit interfacial electron transfer and reduce the bioelectrocatalytic activity between the electrode and bacteria.^51^ Additionally, the inherent hydrophobicity of RGO can reduce its affinity for bacterial attachment.^52^ Thus, the novel strategy of functionalizing RGO with positive charges can solve this problem, strengthening the interaction between bacteria and the cathode and substantially augmenting the biofilm density at the surface of the electrode. The conducting polymer of poly (3,4-ethylenedioxythiophene) (PEDOT) with a positively charged backbone covered on RGO has been constructed and used in a high-performance *E. coli*-driven MFC.^53^ The negatively charged bacteria can easily interact with the positively charged backbone of PEDOT coated on RGO and form a denser biofilm, which can augment the electron transfer. In early research on MES, chemical agents with a positive charge such as chitosan were used to modify the surface of the cathode to reinforce this interaction.^33^ Based on the electrostatic interaction between the bacteria and graphene, RGO was functionalized with positive charges of tetraethylenepentamine (RGO-TEPA), and a carbon cloth cathode was then modified with RGO-TEPA.^54^ Therefore, negatively charged *S. ovata* strains can be easily incorporated into the positively charged matrix of RGO-TEPA through electrostatic interaction *via* a simple self-assembly method (Fig. 5b). The formed 3D intertwined biofilms at the cathode surface can enable faster electron transfer, resulting in better performance of the MES process. The strong electrostatic interaction between the *S. ovata* strains and the positively charged matrix of RGO-TEPA can facilitate electron transportation. The novel *S. ovata* strain called strain met interacted more strongly with RGO-TEPA than the wild-type strain. Due to the stronger bonds with RGO-TEPA, multiple self-assembled spheres were formed in the strain met reactor. The stronger bonds resulted in a high acetate production rate and current consumption. Compared to that of the wild-type *S. ovata*, the acetate production rate increased by 3.3-fold.

Microorganisms with negative surface charges can transfer electrons to non-conductive GO, and GO can then be reduced to RGO through bioreduction.^55,56^ This is the one of the methods for the reduction of GO to RGO. Therefore, bacteria can be involved in catalyzing the reduction of GO to RGO,^57^ and during this process, an RGO-biofilm hybrid electrode can be built. This process can also be called an *in situ* self-assembly method. In an early report, a self-assembled 3D macroporous RGO/bacteria hybrid biofilm on the surface of CC was constructed and used in MFCs.^58,59^ The 3D macroporous RGO/bacteria hybrid can increase the specific surface area, augment the loading of biomass, and form multiplexed conductive pathways. Using a similar approach, the *in situ* self-assembly of a 3D RGO/biofilm was accomplished and used in MES.^60^ Using this *in situ* self-assembly method, a large amount of conductive RGO can be incorporated into the biofilm. Multiplexed conductive pathways were formed, which facilitated electron transfer in the biofilm; thus, the electron transfer rate between the biofilm and the cathode was enhanced.^61,62^ For the microbial reduction self-assembly method, during the reduction process, RGO was intertwined with the bacteria or alternatively covered with *S. ovata* cells. Therefore, the bacterial cells were packaged with the graphene, and were intimately contacted with graphene. Compared to graphene sheets directly covered on the cathodes, the 3D-RGO interlocked networks increased the contact surface area significantly. The larger contact surface can accelerate the highly efficient direct-contact-based extracellular electron transfer between the biofilm and graphene. Therefore, the self-assembled electroactive biofilm can lead to high efficiency of MES for acetate production from CO_2_.

It should be pointed out that the two *in situ* self-assembly strategies are different from each other. In the case of the above-mentioned self-assembly of 3D RGO/biofilm carbon cloth cathodes, upon respiration, the autotrophic microorganisms transfer electrons to GO for the bioreduction of non-conductive GO into conductive RGO. This is a chemical process because of the bacterial participation. However, the self-assembly of the RGO-TEPA-modified cathode structure is *via* an electrostatic interaction process, wherein the negatively charged *S. ovata* strains are incorporated into the positively charged matrix of RGO-TEPA.

In the above-mentioned self-assembly method, it can be seen that in order to improve the interaction with the bacteria, graphene can be doped or functionalized with other functional groups to enhance its solubility and interaction with the bacteria. The self-assembly method also verified that RGO can participate in the biologically active process and has good biocompatibility for promoting the growth and proliferation of electroactive bacteria. RGO can act as a beneficial bridge to connect the bacteria and cathode for electron transport. At the same time, biologically mediated production such as the bacterial reduction of RGO can be used on an industrial scale for graphene-based cathode development in the future.

### 2.3. Chemical methods for graphene-based composite/hybrid fabrication

Using the above-mentioned direct GO reduction and self-assembly method for preparing graphene-based cathodes, only individual graphene-based cathodes have been fabricated. Graphene-based composite/hybrid cathodes can be fabricated by multi-step chemical synthesis methods. We know that the fabrication of graphene with other nanomaterials can produce novel biocompatible composites or hybrids.^63,64^ Due to the additional positive synergistic effect with graphene, nanocomposites or hybrids have superior functional properties than their individual components. Graphene has high biocompatibility and can readily interact with bacterial catalysts. Therefore, graphene-based composites/hybrids can improve the electrical conductivity, enlarge the total surface area, facilitate extracellular electron transfer, and increase the microorganism density. Graphene has been combined with other nanomaterials such as metals, metal oxides, and metal carbides to create composite/hybrid anodes that have been frequently explored in MFCs.^65,66^ These nanocomposites/hybrids show a positive synergistic relationship, exhibiting significant improvements in the MFC properties in all cases.^67–69^ For example, using a two-step chemical method, a TiO_2_/RGO hybrid was constructed and used as an MFC anode. The strong synergistic effect between the good hydrophilicity of TiO_2_ nanocrystals and superior conductivity of RGO contributed to high-performance electrocatalysis.^70,71^ The fabrication of graphene-based composites for MES has been increasing and some promising results have been reported recently.^72^ As a typical example, using a two-step chemical method, a hydrogen evolution catalyst of a Pt nanoparticle-decorated RGO composite was built by Ma *et al.*^64^ Due to the wrinkled surface of RGO coated on the cathode, its area was increased significantly for biofilm attachment. The larger specific surface area of RGO for the distribution of Pt could speed up the activity of the Pt particles. The PtNPs/RGO composite could catalyze the hydrogen evolution reaction rapidly and increase the local H_2_ concentration; therefore, the MES performance was significantly boosted. Using a two-step chemical approach, nanohybrids of MnO_2_/RGO were also constructed by Min's group.^104^ The incorporation of RGO with MnO_2_ further enlarged the surface area of the cathode, and the biocompatibility of RGO was favored the growth of microbes on the electrodes. Due to the combination of the hydrophilicity of MnO_2_ and the high conductivity and high specific surface of RGO, the cooperative effect of the nanohybrid of MnO_2_/RGO efficiently promoted the electron transfer capacity and resulted in high CO_2_ adsorption performance. The typical rippled and crumpled morphology of RGO was decorated with MnO_2_ flowers. The overall morphology of the hybrid looks like many interconnected bunches of flowers. The nanohybrid with the nanowire-flower morphology and RGO network can create more electroactive sites for the microbially catalyzed reaction, which results in an enhanced electron exchange capacity.

## 3. The key roles in electrosynthesis

Modification of the cathode surface is an important approach to improve the interactions between the electrode and microorganisms.^73,74^ To develop high-efficiency cathodes for MES, the material properties should be taken into consideration.^75–78^ Due to its high active surface area, chemical stability, high electrical conductivity, and biocompatibility, graphene is suited to modify cathodes to increase their bacterial loading and boost MES productivity.^79–81^ Graphene-based nanocomposites have far superior functional properties due to the additional positive synergistic effect with graphene, which can improve the extracellular electron transfer for exceptional electrical conductivity and increase the microorganism density due to their high surface area. Graphene-functionalized or graphene-based nanocomposite modified cathodes can increase the electrosynthetic rate of chemicals from CO_2_ and can significantly improve the biofilm density and current consumption. Therefore, graphene and graphene-based nanocomposite bacterial electrodes possess excellent electrochemical properties for high-performance microbial electrosynthesis. Thus, in this section, the key roles of graphene and its nanocomposites in electrosynthesis and their effect on MES performance are discussed.

### 3.1. Augmentation of surface area

**Fig. 6:**
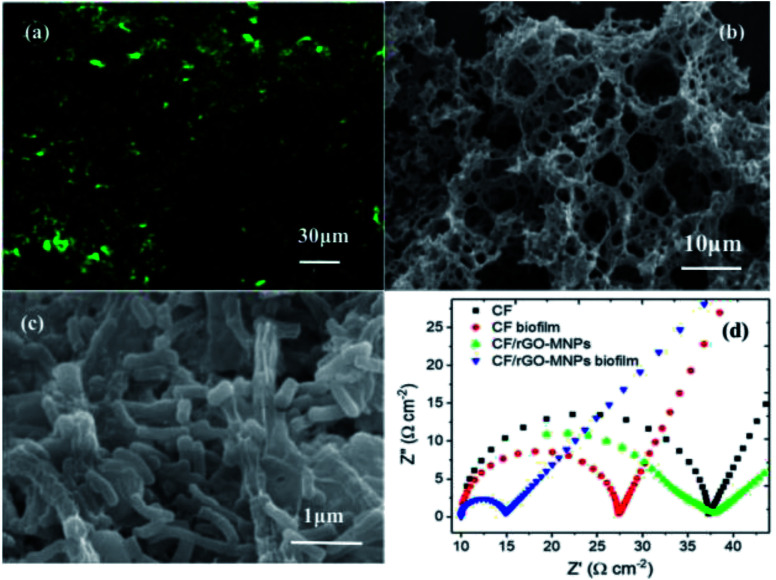
(a) CLSM image of a freestanding RGO paper cathode. (b) FESEM image of CF/RGO NCs. (c) FESEM image of a biofilm for CF/RGO/CC. (d) Electrochemical impedance spectra of reduced graphene oxide with a magnetite nanoparticle-modified electrode (CF/RGOMNPs) and unmodified carbon felt electrode (CF) with or without an attached biofilm in a freshwater medium. (a) Adapted/reproduced from ref. 48 with permission from Springer Nature, copyright 2017. (b and c) Adapted/reproduced from ref. 72 with permission from Elsevier Ltd, copyright 2021. (d) Adapted/reproduced from ref. 73 with permission from IOP Publishing Ltd, copyright 2021.

The high specific surface area of cathodes permits efficient mass transfer within the biofilm and provides sufficient active surface area to increase the bacterial loading. Based on this, recently, many techniques have been utilized to decorate cathodes to augment their surface area, with nanomaterials such as carbon nanotubes and metal oxides. CNTs and other materials have been used in the modification of cathodes to augment the total electrode surface area.^37,82^ The high porosity and high surface area of a CNT-modified nickel hollow fiber cathode fabricated by electrophoretic deposition was reported.^83^ The high specific surface area with superconductive CNTs favored bacterial adhesion, CO_2_ adsorption, a high yield of acetate, and a high electron transfer rate. A highly porous bimetallic oxide of Fe_*x*_MnO_*y*_ microspheres modified on CC as a cathode catalyst was presented.^76^ The high BET surface area (278 m^2^ g^−1^) with a rough surface can provide extra room for microorganisms to colonize; therefore, the bioconversion ability was activated. Graphene modification on the cathode can also improve the available surface area and create more electroactive sites for the microbially catalyzed reaction in MES, resulting in an increased electron exchange capacity with the microbes. The high specific surface area of the graphene-based cathode can maximize the direct mass transfer from the cathode to the microbes for CO_2_ conversion. Taking RGO paper as an example of a cathode, its specific surface area is 5.4 times higher than that of a carbon paper cathode with a similar diameter and thickness.^48^ Thus, the high surface area of the electrode substantially increased the electrode surface area available for electronic interactions with the microbes. This can be confirmed by confocal laser scanning microscopy (CLSM) (Fig. 6a). A dense biofilm of bacterial cells was tightly packed on the RGO paper cathode. The large area of the RGO paper coated by a larger number of bacterial cells indicated that the augmentation of the surface area makes it more compatible for colonization by *S. ovata* in MES reactors, which brought about higher current density and faster acetate production.

Besides the decoration of graphene on the cathodes to increase their total surface area, graphene-based composites or hybrids are also often fabricated and applied to increase the electrode surface area for bacterial loading. A corrugated sheet of graphene with other nanomaterials is more beneficial for bacterial attachment, and the overall high specific surface area of graphene-based three-dimensional composites can supply a sufficient active surface. The mesopores or macropores in graphene-based composites/hybrids favor CO_2_ delivery and adsorption, which can improve the bacterial growth and enhance the conversion rates in MES systems. Nanocomposites of porous copper ferrite/RGO were fabricated and applied in MES.^72^ The overall surface area of copper ferrite/RGO increased significantly, as verified by SEM (Fig. 6b). The high surface area and huge pores of the copper ferrite/RGO structure can make biofilms develop well and enhance the diffusion capability of CO_2_ (Fig. 6c). The copper ferrite/RGO composite can maximize the formation of a continuous electroactive biofilm, increase the electron transfer rate and afford high coulombic efficiencies. Table 1 presents a comparison of the increased surface areas of graphene and graphene-based composite/hybrid cathodes with that of the reference material. From Table 1, we can see that after graphene is introduced, the total area of the novel cathodes increased at least two-fold and the chemical production rate increased greatly compared to that of the control group.

**Table 1:** A comparison of the surface areas of GP/GP-based composite cathodes with that of the reference material

Cathode	Surface area	Chemical production rate	Ref.
GP-CC	2.99 m^2^ g^−1^, 2.2 fold compared to carbon cloth (1.36 m^2^ g^−1^)	6.8	47
RGO paper	0.29 m^2^ g^−1^, 5.40 fold compared to carbon cloth (0.054 m^2^ g^−1^)	8	48
RGO-CuP	1.130 m^2^ g^−1^, 161 fold compared to copper foam (0.007 m^2^ g^−1^)	21.3	84
CF/RGO	158.2 m^2^ g^−1^, 2.24 fold compared to copper ferrite (64.7 m^2^ g^−1^)	1.53	72
MP-RGO	0.824 cm^2^, 2.53 fold compared to carbon felt (0.326 cm^2^)	4.2	73

### 3.2. Reduction of electron transfer resistance

A low charge transfer resistance can speed up electron delivery and bring about higher electron uptake, which can boost the performance of MES. A low charge transfer resistance can result in better electrocatalytic activity due to low charge transfer between the microbes and the electrode. High-conductivity materials in cathodes can promote electron transportation and speed up the extracellular electron transfer capacity, which can achieve higher chemical production. In recent years, in order to reduce the electron transfer resistance, conductive materials or nanocomposites such as conductive polymers, metal sulfides and metal oxides have been commonly used as ideal modification materials to lower the electron transfer resistance. The charge transfer resistance of a cathode coated with the high-conductivity polymer polypyrrole (PPy) was reduced by 33–70% compared to that of the uncoated one.^98^ Molybdenum disulfide (MoS_2_) modified on a CF cathode showed a lower charge transfer resistance (12.1 Ω) than CF (83.3 Ω).^38^ The inexpensive metal oxide nickel ferrite (NiFe_2_O_4_) coated on conventional CF possessed a lower electron transfer resistance (714 Ω) than CF (898 Ω).^94^ In the case of the above-mentioned modified cathodes, not only was there a charge transfer resistance reduction but the current output, acetate production rate, and Faraday efficiency also increased.

Due to its exceptional electrical conductivity, chemical stability, and biocompatibility, graphene can serve as a desirable conductive element between microbes and bacterial electrodes for MES. When graphene is involved in microbial electrochemical systems, its superb electrical properties can speed up electron transfer and improve the overall system properties.^84–86^ Due to the synergetic interaction of nanocomposites compared to their individual components, the improved electrical properties of graphene-based composites or hybrids can facilitate faster electron transfer kinetics.^87–90^ The low charge transfer resistance of graphene or graphene-based nanocomposites/hybrids can also boost the reduction current density and bring about excellent electrochemical properties to enable high-performance MES.^91–94^ In an example using only the introduction of graphene as a cathodes in MES,^60^ the whole-cell resistance was much smaller (12.3 Ω) than that of the control CF cathode (456.8 Ω). The introduction of graphene in the biofilm greatly reduced the cathodic charge transfer resistance and boosted the electron transfer rate. In an example with a nanocomposite of graphene and magnetite, the incorporated graphene reduced the electron transfer resistance and increased the extracellular electron transfer.^73^ The EIS data for an RGO with magnetite nanoparticles (RGO-MNPs) modified electrode and unmodified CF electrode with or without an attached biofilm were plotted as Nyquist curves (Fig. 6d). The charge transfer resistance (*R*_ct_) of the biofilm-enriched CF/RGO-MNPs cathode was low compared to that of the unmodified CF with the biofilm, which verified that it could improve the extracellular electron exchange, resulting in a higher electron uptake and high chemical production in MES. Due to the lower charge transfer resistance, the faradaic efficiency increased two times.

### 3.3. Acceleration of electron transfer and enhancement of bioelectrocatalysis

**Fig. 7:**
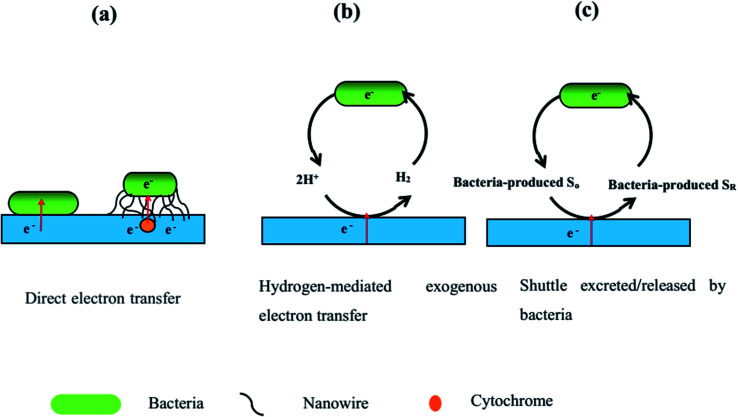
The three possible ways of extracellular electron transfer mechanisms from the cathode to the microbial catalysts. *S*_O_ is oxidized electron shuttle and *S*_R_ is reduced electron shuttle.

Extracellular electron transfer (EET) is a vital pathway to transport electrons between the bacteria and electrodes in MES. According to the literature, EET can be divided into the two mechanisms of direct electron transfer and indirect electron transfer (Fig. 7). Direct electron transfer is when microbial catalysts uptake electrons from electrodes through nanowires or from the cathode through direct contact with the cathode, as shown in Fig. 7a. Indirect electron transfer involves electron transfer through H_2_, formate, Fe(ii) and ammonia as redox mediators or when electrophilic microorganisms release or excrete their own soluble redox mediators to carry electrons from the cathode, as shown in Fig. 7b and c. Electron transfer efficiency is a crucial factor for high-performance MES. Over the whole MES process, microorganisms use the cathode as an electron donor to produce reduced biochemical compounds, making use of CO_2_ as the sole carbon source; therefore, EET is a very important procedure for MES.^95–98^ Accelerating the EET rate is critical for the optimization of MES performance.^99,100^ A high EET rate can result in better carbon dioxide reduction, high biochemical production, high current density, and high coulombic efficiency in MES.^101,102^ The EET rate is associated with the cultivation medium, surface area and roughness of the cathode, interaction between the microbes and cathode, and transfer resistance.^103^ Increasing the interfacial area using a porous three-dimensional scaffold electrode, augmenting the catalytic activity, reducing the electron transfer resistance, and incorporating positively charged species can greatly improve the EET rate.

The high surface area and very thin layer of graphene can favor greater bacterial adhesion and intimate contact, which can increase the direct electron exchange. The functionalization of graphene with positive charges can strength its interaction with the negatively charged bacterial cell surface, which can further increase the EET and significantly speed up acetate production. At the same time, the functionalization of graphene with positive charges can make electrophilic microorganisms release or excrete their own soluble redox mediators, which can bring about indirect electron transfer.^54,73,110^ The high conductivity of graphene can promote electron transfer and further accelerate the EET. The high biocompatibility and permeability of graphene together with its improved bioelectroactive sites can speed up the EET. The synergetic effects of graphene-based nanocomposites can also promote the enrichment of electroautotrophic microbes for biomass clogs, which can reduce the resistance and facilitate the EET. Particularly, the high surface area-to-volume ratio of the porous three-dimensional hierarchical structure of composites based on graphene can further increase bacterial attachment and enhance the microbial EET rate.^104,105^ For example, the fabrication of a novel high-surface-area three-dimensional graphene-nickel foam cathode was reported.^46^ The hierarchical porous graphene-nickel cathode possessed a high specific surface area, which can be beneficial to bacteria entering the inner structure of the 3D electrode to form an electrocatalytically active biofilm. The high conductivity of graphene can form multiplexed conductive pathways, which can expedite electron transfer between the microorganisms and electrode. At the same time, the graphene decorated on nickel foam can be beneficial for hydrogen generation, which can indirectly increase the indirect electron transfer rate and CO_2_ reduction. A volumetric acetate production rate of 3.11 mmol L^−1^ day^−1^ was achieved and 70% of the electrons consumed were recovered.

Due to their unique physicochemical characteristics, graphene and functionalized graphene-based nanocomposites have been developed to promote the accelerated proliferation of electroactive bacteria and their strong interaction with the surface of electrodes. The enlarged surface area, low conductive resistance and high electron transfer rate can boost the capability of MES. Table 2 presents the properties of graphene, graphene-based composites/hybrids and other nanomaterials for MES reported in the last decade. From Table 2, we can see that the incorporation of graphene in cathodes can lead to high-performance MES, with high chemical production, high current density, and high coulombic efficiency.

**Table 2:** MES performance of GP/GP-based composite cathodes and other nanomaterial-based cathodes^a^

Cathode	Production	Production rate	Current densities	CE or FE (%)	Ref.
G-CC	AC	925.5 mMm^−2^ d^−1^	−2450 mA m^−2^	86.5	47
RGO paper	AC	168.5 mMm^−2^ d^−1^	−2580 mA m^−2^	90.7	48
RGO-CF1	AC	2.83 mMm^−2^ d^−1^	−4.9 A m^−2^	77	60
G-NF	AC	3.11 mMm^−2^ d^−1^	−10.2 A m^−2^	70	46
G-CuF	AC	1697.6 mMm^−2^ d^−1^	−21.6 A m^−2^	70.2	84
RGO-TEPA-CC	AC	1052 mMm^−2^ d^−1^	−2358 mA m^−2^	83	54
CF2/RGO	IS, AC	35.37 mgm^−2^ d^−1^	−29.2 A m^−2^	7.78	72
MP-RGO	PHB	91.31 mgL^−1^	−11.7 μA cm^−2^	9.05 (FE)	73
PtNPs/RGO	AC	126.2 gm^−2^ d^−1^	−10 mA cm^−2^	—	64
MnO_2_/RGO	IS, AC	50.07 gm^−2^ d^−1^	−7.8 mA	66.4	104
RGO-WO_3_	AC	5880 mgL^−1^	13.56 ± 0.5 A m^−2^	72	105
Ni–PHF/CNTs	AC	247 mMm^−2^ d^−1^	−332 mA m^−2^	83	83
NanoWeb-RVC	AC	1.3 mMcm^−2^ d^−1^	−3.7 mA cm^−2^	70	37
Fe_*x*_MnO_*y*_-CC	AC	204 mMm^−2^ d^−1^	−6.6 mA m^−2^	58	76
3D Fe_2_O_3_-CC	AC	25.4 mMm^−2^ d^−1^	—	86	75
Chitosan/CC	AC	229 mMm^−2^ d^−1^	475 mA m^−2^	86	33

a AC-acetate, IS-isobutyrate, G-graphene, CC-carbon cloth, CF1-carbon felt, CuP-copper foam, NF-nickel foam, NPs-nanoparticles CF2-copper ferrite, FE-faradaic efficiency, PHB-polyhydroxybutyrate, MP-magnetite nanoparticles, Me-methanol, Ni–PHF-nickel hollow fibers, CNTs-carbon nanotubes, RVC-reticulated vitreous carbon, Fe_2_O_3_-Iron oxide.

In recent years, a vast number of studies have reported that graphene has antibacterial properties.^50,106,107^ However, it needs to be clarified whether the antibacterial properties of graphene come from specific aerobic conditions. Graphene can induce oxidative stress with reactive oxygen species using molecular oxygen. The antibacterial properties of graphene have also been used in water treatment, metal recovery, medical equipment, and tissue engineering scaffolds. However, in the case of graphene in the MES of biochemicals from CO_2_, the growth conditions are usually maintained under strict anaerobic conditions to remove O_2_, with constant gas flushing. In the absence of O_2_, the graphene-coated bioelectrode can promote bacterial proliferation. This is why graphene can exhibit bacterial growth-promoting activity in some reports and antibacterial properties in other reports.

## 4. Perspective and remarks

MES is a promising way to drive the reduction of the greenhouse gas CO_2_ into high-value multicarbon biochemicals using renewable sources of electricity such as solar and wind and effectively store electrical energy in the form of chemical bonds.^108–111^ For purpose of obtaining highly productive MES systems and speeding up their potential scaling up, the low bacterial loading, low electron transfer rate and low productivity of chemicals are great challenges that need to be overcome.^112–114^ High active surface area, highly hierarchical porous networks, and high electronic conductivity in materials are essential to develop highly productive MES systems. Therefore, in this regard, graphene and graphene-based composites can help overcome the challenges and provide opportunities in the future research on MES. Graphene is suited for application as a cathode to increase the bacterial loading and boost MES performance due to its high active surface area, chemical stability, high electrical conductivity, corrosion resistance and biocompatibility. Due to their additional positive synergistic effects over individual graphene, graphene-based nanocomposites have even better functional properties, which can improve the EET and increase the microorganism density due to their exceptional electrical conductivity and high surface area. Therefore, graphene and graphene-based nanocomposite bacterial electrodes possess excellent electrochemical properties for high-performance microbial electrosynthesis. Graphene-modified cathodes increase the electrosynthetic rate of chemicals from CO_2_ and significantly improve the biofilm density and current consumption.

Carbon nanotubes have a high aspect ratio, high surface area, good chemical stability, and excellent electrical conductivity.^115,116^ Carbon nanotubes exhibit high biocompatibility, and can thus promote bacterial metabolism and growth. Therefore, carbon nanotubes have become extremely attractive for applications in MES. In the last decade, some researchers have reported carbon nanotube-based biocathodes for the EMS of chemicals from CO_2_.^36,37,83^ The main approaches for carbon nanotube-based cathode fabrication include electrophoretic deposition (EDP) and CVD. The EDP method is suitable and attractive for large-scale MES-based cathode fabrication. The CVD method is expensive, complicated, and energy and time consuming. We believe that in the near future, graphene has sufficient advantages to tackle the challenges associated with carbon nanotubes in MES. Graphene has a higher specific surface area (theoretical value 2630 m^2^ g^−1^) than carbon nanotubes. Compared with carbon nanotubes, the larger specific surface area of graphene can result in more sites available for microbial interaction and adhesion. The rough surfaces and numerous irregularities in graphene are beneficial for microbial colonization. Graphene has a higher conductivity (10^8^ S m^−1^) than carbon nanotubes. The high conductivity of graphene can promote the electron transportation and speed up the extracellular electron transfer capacity. Due to its easy synthetic process, graphene has a lower fabrication cost than carbon nanotubes. Graphene-based cathode fabrication is inexpensive, simple, and can be up-scaled for industrial-scale production.

Over the last decade, a variety of methods have been developed for designing and fabricating graphene and graphene-based composite cathodes for MES. Although some considerable achievements have been made in this area, such as high current density, high coulombic efficiency, and high chemical production, some problems such as the high cost of graphene, the complicated process, energy/time-consuming process and the small lab-scale synthesis have been encountered, which limit their wide application. The low-cost, high efficiency, pilot-scale, high quality, and high yield synthesis of these products is still a challenge in expanding their fundamental performance and potential practical applications. The key considerations of graphene research are presented as follows:

(i) The EET between microorganisms and cathodes should be further understood through theory and experimentation. Till now, a deeper understanding of the mechanism behind electron transfer and exchange is limited. When graphene is introduced in MES systems, connecting the microbes and cathode, it can accelerate electron transfer and reduce electron transfer resistance; however, there is a lack of knowledge regarding the related EET process. There are no theoretical and experimental studies in this field at present. Determining the electron transfer mechanism for graphene involved in the MES pathway can further result in a major breakthrough and boost the already high EET rate. We believe that with the development of various technical tools, such as electrostatic microscopy, electrochemical surface plasmon resonance, and the establishment of mathematical models, multidisciplinary approaches can shed fresh light on the role of graphene in the EET mechanisms.

(ii) The hydrophobicity of graphene is a big challenge for the large-scale development of MES as graphene has to inevitably come in contact with the electrolyte solution. Hydrophobicity is an intrinsic property of graphene that restricts its dissolution in water, and thus it can inhibit its interaction with bacteria. In order to improve its solubility, graphene is often doped or functionalized with other functional groups such as carboxyl and hydroxy.^117^ However, this reduces the electron mobility and conductivity of graphene. We believe that with the progress of doped and functionalized materials based on graphene, a balance between the wettability and conductivity can solve this problem.

(iii) Although graphene has an outstanding specific surface area, the sufficient utilization of its large specific surface area is another problem. As we know, due to the strong π–π stacking coupled with van der Waals forces between the layers, graphene sheets are prone to restacking and easily form irreversible agglomerates. Therefore, in graphene-based cathode fabrication, the large specific surface area of graphene can be dramatically decreased, which results in an insufficient surface for MES applications. Restraining the agglomeration of graphene sheets and making efficient use of single-layer graphene in MES cathodes should be the focus of further research. As we know, large sheets of single-layer graphene can be grown by the CVD method. Therefore, we can construct a three-dimensional single-layer graphene brush or sponge as an MES cathode. Thus, a three-dimensional graphene brush or sponge can provide a sufficient surface for MES applications.

(iv) The high cost of graphene has become a significant drawback for its expansion, particularly in the competitive commercial market. Graphene and graphene-based composites have been used in MES cathode modification; however, the cost of graphene is higher than that of traditional materials such as carbon cloth and carbon felt, and its chemical production is much more expensive than other methods.^118^ Its production has been substantially increased in the laboratory; however, its larger-scale production has not been exhibited at present. Its high production costs limit its commercialization. The CVD method is expensive, complicated, and energy- and time consuming. The solution-based chemical reduction method can bring about toxic chemical pollution from the GO reduction process. In contrast, mechanical exfoliation can produce graphene on a large scale at low cost and high quality. Thus, in the near future, with the advancement of graphene preparation technology, we forecast that the cost of graphene-based cathode fabrication will decrease and it will be competitive in the market.

(v) The large-scale fabrication of graphene-based cathodes is another critical issue for its industrial-scale production. The small-scale manipulation in the lab, toxic chemicals introduced for the GO reduction process, and complex and time-consuming fabrication process have become shortcomings in their further application. Large-scale graphene-based cathode fabrication is limited by multiple factors such as its inefficient construction strategies, high cost, and low quantity of chemical production in the lab. Industrial-scale graphene-based cathode development and high-quantity chemical production should be integrated with advanced technologies such as the biologically mediated production of RGO and 3D bio-printing.^55,118–120^ With the development of various technologies, we expect the fabrication of large-scale graphene-based cathodes to have a bright future in CO_2_ microbial transformation and utilization.

## 5. Conclusion

Graphene has attracted enormous interest in the fundamental research and potential application of MES due to its suitable electrical and physical characteristics with high affinity for bacterial cells and the formation of high-density electroactive biofilms. In this report, we have briefly reviewed the recent research progress on novel cathodes of graphene and graphene-based composites/hybrids in MES systems. We have emphasized the strategies behind the design and fabrication of graphene-based cathodes with their key roles in electrosynthesis, and have included our perspective and remarks as well. This review not only provides an updated progress on the fabrication of all kinds of MES cathodes based on graphene and its composites but also demonstrates their important roles in the augmentation of the surface area, reduction in the resistance and increase in the electron transfer rate. The comprehensive discussion on the fabrication techniques and critical function of these cathodes in MES can provide fundamental insight into understanding and designing graphene-based cathodes for their further optimization and improvement. Due to the research progress on graphene and related technologies, it can be expected that graphene will play a significant role in the further development of high-efficiency MES and can speed up the commercialization process.

## Conflicts of interest

There are no conflicts to declare.

## Supplementary Material
